# Guided online self-management interventions in primary care: a survey on use, facilitators, and barriers

**DOI:** 10.1186/s12875-016-0424-0

**Published:** 2016-03-09

**Authors:** Rosalie van der Vaart, Vera Atema, Andrea W. M. Evers

**Affiliations:** Unit of Health, Medical and Neuropsychology, Leiden University, Leiden, The Netherlands; Department of Psychiatry, Leiden University Medical Centre, Leiden, The Netherlands

**Keywords:** Primary care, Mental care, Online interventions, Self-management, Implementation

## Abstract

**Background:**

Guided online psychological self-management interventions offer broad prospects for the treatment of people with mild to moderate mental health problems, but implementation is challenging. The aims of this study are (1) to gain insight into use of and intention to use these interventions among primary care health professionals, (2) to determine the main barriers to use such interventions among non-users.

**Methods:**

An online survey based on the Unified Theory of Acceptance and Use of Technology (UTAUT) was disseminated among mental health counsellors (MHCs; in Dutch POHs) in GP practices and primary care psychologists (PCP) in mental health care practices. The survey covered the current use of online interventions, the intention to use these in the future, and an operationalization of the UTAUT concepts: performance expectancy, effort expectancy, social influence, and facilitating conditions.

**Results:**

In total, 481 MHCs and 290 PCPs responded (24 %). Of them, 49 % of MHCs and 21 % of PCPs currently use online interventions in their treatments. A further 40 % of MHCs and 27 % of PCPs plan to introduce such interventions within the next year. Both groups were moderately positive about the presence of eHealth facilitators in their daily practice. Among current non-users, performance expectancy and facilitating conditions were significant predictors of usage intention in both groups of health professionals.

**Conclusions:**

Use of and intention to use online interventions is relatively high in Dutch primary care. Non-users, particularly, experience several barriers which need attention to enhance implementation. There is a need for further efforts regarding facilitation of and education on eHealth, as well as for research directed to its normalization in daily practice.

**Electronic supplementary material:**

The online version of this article (doi:10.1186/s12875-016-0424-0) contains supplementary material, which is available to authorized users.

## Background

The use of information and communication technology (ICT) in health care (eHealth) is evolving rapidly. ICT is used both to support regular care and care systems and as a means to actually provide care online. eHealth offers prospects for regulating the demand on health care, which is growing due to the aging population, the increase in chronic diseases, and higher treatment rates due to better diagnostics and more widely available effective interventions. Where mental health care is concerned, integrating health services into primary care is increasingly considered the most viable way to respond to this growing demand and to ensure broad access to mental health care [1]. In the Netherlands, primary care physicians are therefore urged to adopt an increasing role in treating people with mild to moderate mental health problems and to refrain from referring patients to specialized mental health services as much as possible. This system is based on a stepped care principle, which aims to provide the best fit between the severity of a patient’s problems and the intensity of the treatment he or she receives [2].

Guided online psychological self-management interventions (often based on cognitive behavioural therapy principles [3]) offer good prospects for the treatment of people with mild to moderate mental health problems in an accessible manner. These web-based interventions tend to combine the technological possibilities of online programs with the tailored approach of face-to-face interventions, resulting in a larger scalability with lower costs per additional user [4]. Numerous studies and systematic reviews have shown these online interventions to be effective in both primary and secondary care, for a broad range of problems such as mood disorders, anxiety, and adaptation to chronic somatic conditions [5–9]. Generally patients follow a set of modules (often consisting of, for instance, psycho-education, assignments, registrations, and relaxation training), while they receive feedback and coaching from a therapist through online messages. In this way, people with a non-severe or non-complex diagnosis can, in a relatively short time and with limited assistance, learn essential skills to self-manage their problems.

In spite of the great potential of online interventions and their value for alleviating the pressure on mental health care, the adoption of new forms of care is complex, especially when it comes to eHealth [10–12]. In implementing health technologies, a variety of economic, political, and organizational factors are key. In the Netherlands, policy changes on these matters are becoming established, and the use of eHealth in primary care has been being stimulated, largely top down, for some years now [13]. This means that eHealth technology is becoming increasingly available, and health care professionals are encouraged to use such interventions. However, this brings about a variety of implementation challenges on the level of the individual professional and the social context in which they work; health professionals need to learn new skills and alter the way they work to incorporate online care into their daily routines. These factors are represented in the Unified Theory of Acceptance and Use of Technology (UTAUT) by Venkatesh et al. [14]. This model provides an overview of aspects that play a large role among individuals when implementing health technology. This model shows that the efforts that can be expected to be invested and the benefits that could be gained from technology are essential predictors for the behavioural intention to use health technology. Furthermore, the social influence and facilitating conditions experienced on the work floor can also have significant influence on the success of a new technology.

In order to gain insight into the implementation of guided online psychological self-management interventions in primary care, this paper studies the extent to which they are actually used in routine clinical practice by mental health counsellors (MHC) working in GP practices, and by primary care psychologists (PCP). These two groups of health professionals both deliver primary care, but in a different context and with a different treatment intensity [13]. The MHC is an official collaborative position within a GP practice. These health professionals are mostly trained as psychologists or psychiatric nurse practitioners, and are governed by the GP or seconded from larger mental health care institutions. They offer short-term counselling to patients with mild psychological problems (with or without DSM diagnosis). A PCP on the other hand treats patients with mild to moderate problems (a DSM diagnosis is required); these patients are referred to the PCP by the GP. If these forms of primary care prove insufficient, the patient can be referred to specialized mental health care in secondary care. Both groups of primary care professionals could benefit from using online interventions, but they might use such interventions differently due to the nature of their care and the type and severity of problems they encounter.

The present study used an online survey among these two large groups of mental health professionals to gain insight into their current use of technology and online interventions and the behavioural intention to use them. The study used the UTAUT model to investigate the main facilitators and barriers among non-users of online interventions, in order to map essential variables that could enhance successful implementation.

## Methods

### Participants and procedure

Both groups of participants were recruited by (personal) e-mail. The MHCs were selected using a national health care portal (https://www.zorgkaartnederland.nl/) which provides an overview of all general practitioners in the Netherlands. Via this portal, websites of GP practices were checked to find the names and e-mail address of MHCs. If it was unclear which individual held the position of MHC, or if no e-mail address was found, the practice was contacted by phone to ask for the required information. This resulted in 1655 e-mail addresses. In addition, several national mental health care institutions which were known to second MHCs to GP practices were approached and asked to forward the survey invitation to all their practising MHCs. To recruit the PCPs, the website of the Dutch national association of independent psychologists (LVVP) was used, which presented the contact information of all associated primary care psychologists. This resulted in 1332 e-mail addresses. Additionally, Google was used to find further PCPs: using the search terms “primary care psychologist” and “psychologist basic health care” (*“eerstelijnspsycholoog*” and *“psycholoog gezondheidszorg basis*” in Dutch). This led to an additional 371 addresses. In the first mailing, some e-mails failed to deliver, for reasons such as addresses that no longer existed, respondents that were no longer employed at that practice, or out-of-office replies that reported the addressee would not return before the closure-date of the survey. Two weeks after the first mailing a reminder e-mail was sent to all known addresses. In total, 1601 MHC and 1641 PCP addresses were contacted. In a few cases these may not have been unique addresses, since it is possible that the same health professional was e-mailed at multiple addresses (if employed at several institutions). Some may also have received the survey via multiple routes (since it was also distributed to larger groups of professionals, for instance when sent to a practice e-mail address).

The e-mail contained a personal invitation addressed to the health professional by name (when known); the e-mail included an information letter and a link to the online survey in Qualtrics (2015 Qualtrics, LLC). The information letter explained the purpose of the study and its voluntary nature, the use and anonymization of the data, the estimated time needed to participate (10 min), and the reward for participating in the study (four gift certificates of €50 were raffled among the participants). The online survey itself also contained this information letter and an informed consent form. Not responding to the survey or opting out in the informed consent form were considered as choosing not to participate in the study. MHCs who work at several GP practices were asked to fill in the questionnaire with reference to the GP practice in which they were working at that time. The study was approved by the Psychology Research Ethics Committee (PREC) of Leiden University.

### Questionnaire

The study consisted of a questionnaire in two versions: one for the MHCs and one for the PCPs. The questionnaires were virtually identical, with the exception of detailed questions on the respondent’s professional background and workplace.

The questionnaire (see ‘Additional file 1’) consisted of three parts: (I) background information about the health care professional and the practice; (II) general and practice-related use of the internet, and (III) experience with using and/or intention to use online psychological self-management interventions. The background information of the health professional included gender, age, professional background, and hours employed. The background information about the health care practice included number of staff, average number of consultations per patient and time invested per consultation, and characteristics of the care mostly provided. In part two, internet use and experience with online care was measured; this included the presence of different types of eHealth technologies already in use at the practice.

Measurement of experience with using and/or intention to use online psychological self-management interventions was based on the UTAUT, including performance expectancy, effort expectancy, social influence, facilitating conditions, and behavioural intention. All subscales were measured with three to eight items formulated as statements. Participants could answer these statements on a 5-point Likert scale, ranging from completely disagree (1) to completely agree (5). An overview of the subscales, including the number of items and example items, is shown in Table 1. The subscale behavioural intention included one deviating item regarding the current use of online interventions and the timeframe in which a respondent expected to start using online interventions (“How soon do you expect to use online guided self-management programs?” with answer options ranging from “I use them already” to “Never”). A complete overview of all the items per subscale can be found in Additional file 1.

**Table 1:** Subscales, number of items, and example items used in the UTAUT survey

Subscale	No. of items	Example item
Performance expectancy	8	I expect/perceive that online psychological self-management interventions will be effective for our patients
Effort expectancy	7	I expect/perceive that guided online psychological self-management interventions require a lot of new skills
Social influence	3	I expect/perceive that online psychological self-management interventions will be used a lot by my colleagues
Facilitating conditions	6	I expect/perceive that online psychological self-management interventions match with the current state of technology in my practice
Behavioural intention	3	I intend to use/continue using online self management programs in my work.

### Data analysis

Analyses were performed using the Statistical Package for the Social Sciences (IBM SPSS Statistics 21). Scale scores were calculated and Cronbach’s alphas of the internal reliability were analysed. Descriptive statistics were performed to describe the study sample, their internet experience, and their scores on the UTAUT items. Chi-square tests and t-tests were applied as appropriate to analyse whether reported differences between the two groups of health professionals and between users and non-users within these groups were significant.

To gain insight into the constructs that predict behavioural intention among non-users, multiple regression analyses were performed. Since the distribution of scores on the scale ‘behavioural intention’ deviated from normality, non-parametric Spearman’s correlations were calculated to determine the relationship between the UTAUT variables performance expectancy, effort expectancy, social influence, and facilitation conditions, and the dependent variable behavioural intention. Based on the statistical significance of these relations, multiple regression analyses were carried out to examine the further nature of these relations for both groups.

## Results

### Study samples

The response of completed surveys was 481 among the MHCs and 290 among the PCPs. Due to the method of data collection, it is not possible to calculate a precise response rate. However, 3242 e-mail addresses were approached, which provides an estimated response rate of 24 %. Table 2 shows the characteristics of the two groups and the health care practices in which they worked. Most respondents were female (76 % in both samples), and the MHCs were slightly younger than the PCPs (*M* = 45 and 52 respectively). Most of the MHCs had a professional background as a psychiatric nurse practitioner (36 %) or psychologist (25 %). Most PCPs had a background as a health care psychologist (86 %; which is a specialized qualification for working as an independent psychologist in the Netherlands) and/or a psychotherapist (33 %). In both disciplines respondents mostly worked part-time (*M* = 22 and 25 h respectively). Many of the MHCs worked in more than one practice (60.5 %, data not shown in Table). As regards the health care practices in which the respondents worked, most MHCs worked in GP practices with 4 or more GPs (55 %, data not shown in Table) and one other MHC colleague (*M* = 1.2; *SD* =1.78). Most PCPs worked in a practice with two other colleagues (*M* = 2.3; *SD* = 3.7). In the MHC practices, most patients were seen for 4–5 sessions, which took on average 36 min each. The PCPs generally saw their patients for a longer period of time, on average 6–10 sessions of 55 min.

**Table 2:** Background information about the professional and the practice (*N* = 771)

Characteristics	MHC	PCP
	*n* = 481 (%)	*n* = 290 (%)
Health professional		
Gender		
Female	363 (75.5)	221 (76.2)
Male	118 (24.5)	69 (23.8)
Age (M, SD)	45 (11.1)	52 (9.8)
Professional background^a^		
Psychiatric nurse practitioner	174 (36.2)	n.a.
Social worker	51 (10.6)	n.a.
Psychologist, MSc.	122 (25.4)	11 (3.8)
Health care psychologist (GZ-psycholoog)	n.a.	250 (86.2)
Psychotherapist	n.a.	96 (33.1)
Other (pedagogue, nurse, counsellor)	134 (27.9)	45 (15.5)
Hours employed on weekly basis (M, SD)	22 (9.4)	25 (12.3)
Number of peer colleagues in the practice (M, SD)	1.2 (1.78)	2.3 (3.70)
Health care practice		
Number new patients each month		
< 20	231 (48.0)	92 (31.7)
20–40	211 (43.8)	89 (30.7)
> 40	39 (8.1)	109 (37.6)
Number of consultations during one complete treatment		
1–3	29 (6.0)	10 (3.4)
4–5	284 (59.0)	12 (4.1)
> 5	168 (34.9)	n.a.
6–10	n.a.	197 (67.9)
> 10	n.a.	71 (24.5)
Number of minutes spent per therapy session (M, SD)	36 (9.9)	55 (10.6)

^a^multiple types of registrations can be valid at the same time

With regard to the characteristics of the care provided, MHCs reported mostly treating patients with stress/burn-out (91 % of health professionals reported seeing patients with these problems), mood disorders (88 %), and anxiety (62 %). PCPs also saw a large number of patients who suffered from these types of problems: anxiety (98 %), mood disorders (97 %) and stress/burn-out (88 %). However, they also reported seeing patients with a variety of other problems, such as fatigue (74 %), problems in a social context (73 %), coping with somatic symptoms and chronic diseases (66 %), sleeping problems (66 %), and pain (55 %). The type of care the MHCs provided for their patients was mostly clarification of the problem and diagnostics (81 %), interventions/treatment (70 %), psycho-education (68 %), and coaching patients in self-management (61 %). The main focus in care provision among PCPs was on interventions/treatment (98 %), followed by clarification of the problem and diagnostics (71 %), and psycho-education (54 %).

### Internet experience

Almost all respondents used the internet very frequently (see Table 3), and rated their internet skills as good to very good (79 and 77 % for MHCs and PCPs, respectively). All respondents used the internet in their professional setting, mostly to search for information on referrals, medical information or information on insurances and reimbursements, but also to communicate with patients via e-mail (67 and 94 % respectively). Concerning the availability of eHealth technology in the workplace the majority of the respondents stated that electronic medical records were available (88 and 71 %), and most also had a website with information for patients (63 and 86 %). Online self-management modules were available at 36 % of the MHC practices and 21 % of the PCP practices surveyed.

**Table 3:** General and practice-related usage of the internet (*N* = 771)

Internet usage	MHC	PCP
	*n* = 481 (%)	*n* = 290 (%)
General internet usage		
(Virtually) every day	437 (90.9)	282 (97.2)
Several times a week	31 (6.4)	6 (2.1)
1 day a week to (virtually) never	13 (2.6)	2 (0.7)
Subjective internet skills		
Good-very good	379 (78.8)	222 (76.6)
Average	76 (15.8)	57 (19.7)
Poor-very poor	26 (5.4)	11 (3.8)
Work-related internet usage	481 (100.0)	290 (100.0)
Searching for referral information	456 (94.8)	243 (83.8)
Searching for medical information	433 (90.0)	245 (84.5)
E-mail communication with patients	329 (68.4)	272 (93.8)
Searching for insurances and reimbursements	142 (29.5)	211 (72.8)
Available technologies in health care practice		
Electronic medical records	424 (88.1)	205 (70.7)
Website with patient information	304 (63.2)	236 (86.1)
Online self-management modules	175 (36.4)	62 (21.4)
Webportal with patient records	158 (32.8)	31 (10.7)
eConsult	136 (30.1)	66 (22.8)
Electronic/online screening	129 (26.8)	91 (31.4)
Online appointment tool	133 (23.5)	54 (18.6)

### Experience with online psychological self-management interventions and intention to use it

A substantial number of both MHCs and PCPs had experience with online psychological self-management interventions (as is shown in Table 4). Almost 60 % of the MHCs and almost a third (29 %) of the PCPs had used them in their treatments. 49 % of the MHCs and 21 % of the PCPs reported that they were currently using online psychological self-management interventions in their treatments. A further 40 % of the MHCs and 27 % of the PCPs planned to use them within the next year. Among the PCPs, a substantial proportion of the respondents expected to be using online interventions within 2–5 years (32 %). Chi-square and t-tests show that the differences between the two groups as regards experience of using and intention to use online psychological self-management interventions are significant. Further, the intention among current users to continue using online interventions is significantly higher than the intention among non-users to start using online interventions.

**Table 4:** Experience with online psychological self-management interventions and intention to use them (*N* = 771)

Experience and intention	MHC	PCP	*P* ^a^
	*n* = 481 (%)	*n* = 290 (%)	
Experience			
Has seen such interventions	420 (87.3)	194 (66.9)	.000
Has been trained to use such interventions	303 (63.0)	105 (36.2)	.000
Has used such interventions in treatments	287 (59.7)	85 (29.3)	.000
Usage			
Currently uses online interventions in treatment	234 (48.6)	62 (21.4)	.000
Expected time frame of usage among non-users			.000
Within the next 6 months	89 (18.5)	26 (9.0)	
Within the next year	103 (21.4)	52 (17.9)	
Within 2 to 5 year	41 (8.5)	92 (31.7)	
Not within the next 5 year	6 (1.2)	28 (9.7)	
Never	6 (1.2)	28 (9.7)	
Missing	2 (0.4)	2 (0.7)	
Intention to use (α = .95 for MHC and .96 for PCP)			
In the complete sample (M, SD)^b^	4.18 (.88)	3.31 (1.20)	.000
Among current users (M, SD) (*n* = 296)^c^	4.53 (.66)	4.18 (.80)	.003
Among current non-users (M, SD) (*n* = 472)^c^	3.86 (.94)	3.07 (1.18)	.000

^a^Chi-square test for experience (dichotomous) and t-tests for intention

^b^Measured with three statements using a 5-point Likert scale with scores (1) totally disagree, (2) partly disagree, (3) neutral, (4) partly agree, and (5) totally agree

^c^Differences on intention to use between users and non-users differ significantly, with *P* < .001 in both groups

### Facilitators and barriers

Table 5 shows the scores on the UTAUT subscales and their Cronbach’s alphas, which are satisfactory. It can be seen that on average the MHCs surveyed tended not to fully agree with the statements. The PCPs on average gave a neutral response: they neither agreed nor disagreed. On performance expectancy, social influence, and facilitating conditions, the means differ significantly between the groups, with the MHCs scoring higher. Looking at the differences between users and non-users of online interventions, it can be seen that users structurally reported higher scores on all the UTAUT constructs than non-users; this applies to both MHCs and PCPs.

**Table 5:** Scores on the UTAUT constructs (*N* = 771)

Construct^a^	MHC		PCP		*P* ^c^
	α	*n* = 481 (M, SD)	α	*n* = 290^b^ (M, SD)	
Performance expectancy	.86	3.69 (.66)	.88	3.18 (.78)	.002
Users^d^		3.86 (.58)		3.56 (.78)	.007
Non-users		3.54 (.70)		3.07 (.75)	.000
Effort expectancy	.71	3.46 (.59)	.68	3.18 (.57)	.15
Users		3.62 (.55)		3.41 (.60)	.01
Non-users		3.32 (.60)		3.12 (.54)	.000
Social influence	.69	3.43 (.72)	.71	2.93 (.85)	.000
Users		3.58 (.67)		3.22 (.89)	.01
Non-users		3.30 (.75)		2.85 (.87)	.000
Facilitating conditions	.79	3.65 (.68)	.83	3.07 (.83)	.000
Users		3.86 (.55)		3.50 (.85)	.006
Non-users		3.45 (.73)		2.95 (.79)	.000

^a^All constructs were measured with statements using a 5-point Likert scale with scores (1) totally disagree, (2) partly disagree, (3) neutral, (4) partly agree, and (5) totally agree

^b^ *N* = 242 for Social influence and 252 for Facilitating conditions; since not all items in these scales were applicable for PCPs, the scale score could not be calculated for all respondents

^c^T-tests

^d^Differences on all the UTAUT constructs differ significantly between users and non-users, with *P* < .01 in both groups

When studying the relationships between the UTAUT concepts and non-users’ intention to use online psychological self-management interventions in the future, all UTAUT concepts showed significant correlations (mostly moderate to high) among both the MHCs and PCPs surveyed (Performance expectancy rho = .66 and .64; Effort expectancy rho = .56 and .40; Social influence rho = .42 and .46; and Facilitating conditions rho = .62 and .68 respectively for MHCs and PCPs, all with *P* < .001).

Multiple regression analyses using the enter method showed that the UTAUT model explained 59 % and 57 %, respectively, of current non-users’ behavioural intention to use online psychological self-management interventions (MHC: F(4, 244) = 84.518, *p* < .000, *R*^*2*^ = .59, *R*^*2*^_Adjusted_ = .58; PCP: F(4, 177 = 57.805, *p* < .000, *R*^*2*^ = .57, R^2^_Adjusted_ = .56). Performance expectancy and facilitating conditions were found to be significant predictors among both the MHCs and the PCPs surveyed. Effort expectancy was only a significant predictor among the MHCs (see Table 6).

**Table 6:** Results of multiple regression analyses of UTAUT variables on behavioral intention

	MHC			PCP		
	*B*	SE *B*	β	*B*	SE *B*	β
Performance expectancy	.61	.09	.46*	.62	.12	.40*
Effort expectancy	.23	.08	.15**	−.18	.14	−.08
Social influence	−.08	.08	−.07	.10	.09	.07
Facilitating conditions	.39	.10	.31*	.64	.13	.42*

*p < .000

**p < .01

## Discussion

Guided online self-management interventions have a high potential to enhance psychological care in primary care; however, the implementation of these interventions is often complex. The primary aim of this study was to investigate to what extent these interventions are used among mental health professionals in primary care. The results showed that health technologies in general are quite integrated into clinical practice, as many health professionals use such technologies to search for information, communicate with their patients, and keep medical records. Among the health care professionals surveyed, online self-management interventions are currently used by almost half of the mental health counsellors in GP practices and by a fifth of primary care psychologists in independent mental health care practices. These numbers are high in comparison with previous studies, which have shown that the take-up of online care in clinical practice is often disappointing [3, 9, 15]. Moreover, the intention that both groups report to use guided online psychological self-management interventions in the future seems positive, especially among MHCs. In this group, 40 % expect to use online interventions within the next year. With 49 % already using them, this leaves only 11 %, many of whom expect to use online interventions in a more distant timeframe of 1–5 years. Among the PCPs, the group of those who not expect to use online interventions in the near future is larger: more than half report that this will take at least 2 years.

The second aim was to study the facilitating and inhibiting factors associated with use of and intention to use guided online psychological self-management interventions; here some significant differences were found between the two groups of health professionals. MHCs more often reported agreeing that the facilitating conditions for the use of online interventions in their practice were sufficient, that the use of online interventions matched their current way of working, and that their management encouraged them to use such interventions. In both groups this factor was shown to be an essential predictor for the behavioural intention among non-users to start using the interventions in the future. This might explain the differences between MHCs and PCPs, both in current use and expected use, since GPs are actively encouraged to use eHealth applications in Dutch policy. Furthermore, a difference in social influence was found, which shows that the two groups of health professionals have differing perceptions of how positively their colleagues and organizations view online interventions. PCPs often work more independently (which is reflected in a lower response rate on the items of social influence) and therefore might experience less encouragement or support from fellow health professionals.

Another difference was found in performance expectancy, which also proved an important predictor of intention in both groups of non-users. MHCs were more positive on the usefulness they expected (or already noticed) of online interventions in terms of perceived effectivity, usefulness in their patient population, enhancement of quality and diversity of care, and the increase of their own productivity. A possible explanation for this difference lies in the patient population of the two groups. MHCs may see online interventions as a valuable addition to their current care provision, to use them for psycho-education and support of patients with mild problems. There are many primary prevention interventions -on promoting self-help with mental problems, coping with somatic diseases, or adapting one’s lifestyle, for instance- which could all be very usable in a GP practice as a first step in treatment. PCPs, on the other hand, regularly see people with moderate mental health problems, for which they might deem online treatment unsuitable. While research on online CBT shows that it is effective for patients with more severe problems [5–7], previous research has also shown that health professionals are hesitant about the suitability of online care for their patients. A study by Hermens et al. [16], for example, showed that health professionals reported that they were uncertain about the appropriateness of eHealth interventions for depressed patients and about how to ensure a working relationship with a patient during an eHealth intervention.

Although these findings show that online interventions are quite widely available in Dutch primary care, the results also correspond with studies on the core challenges of eHealth implementation. The scores on the UTAUT factors, which reflect the extent to which health professionals agree that facilitators are present, show that health professionals who are not yet using online interventions are somewhat hesitant about implementation of these interventions. Facilitating conditions, performance expectancy, and effort expectancy proved to be predictors of behavioural intention among non-users. In other studies too these predictors emerged as a key issue in ensuring successful implementation [17–19]. The scores on UTAUT concepts do not reflect a great deal of optimism among current users either, which might raise questions on how health professionals actually use online interventions in treatment, and to what extent such interventions are part of daily practice, even when they are available. Murray et al. [20] show in a qualitative study that it is essential for health professionals to feel that health technologies have a positive impact on their interactions with patients, and match their skills and the goals of their organization. When making the step to implementation in practice, more research is needed on whether and why interventions are effective and useful, and for whom, when, and how they can best be used [10, 16, 21]. Furthermore, uptake and normalization should be investigated, with a focus on acceptance and use of, and attitudes to online interventions, to tackle the follow-up challenges in the implementation of online interventions in clinical practice [22–24].

In this study a very broad sample of both MHCs and PCPs were invited to participate, and the recruitment database covered a large part of the entire population of both groups. Nevertheless, it should be taken into account that a response bias could be present in the final sample. The estimated response rate is 24 %, which is quite normal for such a large survey study. Still, by sending out one more reminder, we could have increased the number of responses [25]. Furthermore, it is possible that health care professionals who are already interested in eHealth or working with online interventions might be more inclined to fill in the survey, resulting in a somewhat overly optimistic view. Also, respondents could have answered the questions in a socially desirable manner, since the government stimulates health professionals to use eHealth technologies. This might also explain the higher response rate among MHCs, who are expected to deliver a certain percentage of their care via online technologies. No data is available on non-responders, so it is impossible to examine to what extent responders and non-responders differ.

To enhance the use of online interventions in clinical practice and to overcome barriers concerning performance and effort expectancy, more attention could be paid to the competences and skills of health professionals. Education in online care should become a structural part of professional training (both initial training and post- and in-service training), to teach health care professionals what online care entails and how they can use it in daily practice [26, 27]. This also includes e-therapy skills to learn how to communicate with patients via online media and empower patients to self-manage their treatment [28].

## Conclusion

From these results it can be concluded that guided online psychological self-management interventions are widely available in Dutch primary care, especially in GP practices. Almost 90 % of the mental health counsellors in GP practices and almost half of the primary care psychologists in independent mental health care practices surveyed already use these kind of interventions or have the intention to do so within the next year. As regards the UTAUT factors, both groups are moderately positive about the presence of facilitators in their daily clinical practice. Performance expectancy, effort expectancy, and facilitating conditions are significant predictors of the intention to start using online interventions in the future. Further effort and research is essential to enhance these factors and to focus on the acceptance and normalization of online interventions in daily practice.

## Additional file

Additional file 1: Contains the questionnaire that was used for this study. (DOCX 37 kb)
