# Supplementary material for: Guided online self-management interventions in primary care: a survey on use, facilitators, and barriers
Source: BMC Fam Pract. 2016 Mar 9;17:27. doi: 10.1186/s12875-016-0424-0 (PMC4785635; doi:10.1186/s12875-016-0424-0)
Supplement: Additional file 1: — Contains the questionnaire that was used for this study. (DOCX 37 kb) [file 12875_2016_424_MOESM1_ESM.docx]

**Additional file 1: Survey**

*U and your practice*

| 1. | What is your age? | …….. years |
| --- | --- | --- |
| 2. | What is your sex? | □ Male |
|  |  | □ Female |
| 3. | What is your professional background? | □ Psychiatric nurse practitioner |
|  |  | □ Social worker |
|  |  | □ Psychologist, MSc.  □ Health care psychologist  □ Psychotherapist |
|  |  | □ Other, namely…………………………. |
| 4. | How many hours are you employed on a weekly basis? | ……. hours |
| 5. | How many peer colleagues do you have at your practice? | ……. (number of colleagues) |
| 6. | How many new patients do you see every month, on average? | □ less than 20 |
|  |  | □ 20 to 30 |
|  |  | □ 30 to 40 |
|  |  | □ More than 40 |
| 7. | How much time do spent per session, on average? | ……… min |
| 8. | How much sessions do you spent per patient, on average? | □ 1 to 3 |
|  |  | □ 4 to 5 |
|  |  | □ 6 to 10 |
|  |  | □ More than 10 |
| 9. | With what reason/referral indication do patients mostly come to your practice? (max 3 answers). | □ Mood problems |
|  |  | □ Anxiety |
|  |  | □ Stress/burn-out problems |
|  |  | □ Addiction problems |
|  |  | □ Sleeping problems |
|  |  | □ Pain |
|  |  | □ Fatigue |
|  |  | □ Sexual problems |
|  |  | □ Coping problems concerning (chronic) somatic conditions |
|  |  | □ Social problems |
|  |  | □ Other, namely ………………………………………… |
| 10. | What type of help do you mainly offer your patients? (max 3 answers) | □ Signalling |
|  |  | □ Problem clarification and diagnostics |
|  |  | □ Psycho-education |
|  |  | □ Self-management coaching |
|  |  | □ Intervention/treatment |
|  |  | □ Relapsprevention |
|  |  | □ Other, namely……………..……………… |

*Your, your practice and technology*

| 11. | How often do you use the internet  (in general, i.e. to email or to search for information) | □ (Virtually) every day |
| --- | --- | --- |
|  |  | □ Several times a week |
|  |  | □ About 1 day a week |
|  |  | □ Less than 1 day a week |
|  |  | □ (Virtually) never |
| 12. | How would you rate your internet skills? | □ Very good |
|  |  | □ Good |
|  |  | □ Average |
|  |  | □ Poor |
|  |  | □ Very poor |
| 13. | Do you use the internet for work?  If yes, please indicate for what purpose. (you can check multiple boxes) | □ No, never |
|  |  | □ Yes, to search for medical information |
|  |  | □ Yes, to search for referral information |
|  |  | □ Yes, to search for information on insurances and reimbursements |
|  |  | □ Yes, to communicate with patients via e-mail |
|  |  | □ Other purpose, namely………………………………… |
| 14. | What technology is already available in your practice? (you can check multiple boxes) | □ Electronic medical records |
|  |  | □ Website with patient information |
|  |  | □ Electronic/online screening |
|  |  | □ Webportal for patients to access their records |
|  |  | □ Online appointment tool |
|  |  | □ eConsult (encripted e-mail) |
|  |  | □ Tele-consult |
|  |  | □ Online self-management modules |
|  |  | □ None of the above |
|  |  | □ Other, namely …………………………………………… |

*You, your practice and online self-management*

| 15. | Have you ever seen an online self-management program? | □ Yes |
| --- | --- | --- |
|  |  | □ No |
|  |  | □ I don’t know |
| 16. | Have you ever been trained to use an online self-management program? | □ Yes |
|  |  | □ No |
|  |  | □ I don’t know |
| 17. | Have you ever used an online self-management program? | □ Yes |
|  |  | □ No |
|  |  | □ I don’t know |

18. I expect/perceive online self-management programs to …*

|  |  | Totally disagree | Partly disagree | neutral | Partly agree | Totally agree |
| --- | --- | --- | --- | --- | --- | --- |
| [PE] a. | …be effective for my patient population. | □ | □ | □ | □ | □ |
| [EE] b. | …be easy to use. | □ | □ | □ | □ | □ |
| [PE] c. | …be useful for my patient population. | □ | □ | □ | □ | □ |
| [PE] d. | …increase the quality of my care provision. | □ | □ | □ | □ | □ |
| [EE] e. | …ask a lot of practice and schooling. | □ | □ | □ | □ | □ |
| [PE] f. | …increase the diversity of my care provision. | □ | □ | □ | □ | □ |
| [FC] g. | …fit within the financing structures of my practice. | □ | □ | □ | □ | □ |
| [EE] h. | …are time consuming to use. | □ | □ | □ | □ | □ |
| [FC] i. | …fit within the technological circumstances of my practice. | □ | □ | □ | □ | □ |
| [PE] j. | …increase the productivity of my practice. | □ | □ | □ | □ | □ |
| [SI] k. | …be seen as a positive development among my colleagues. | □ | □ | □ | □ | □ |
| [PE] l. | …increase the interaction/communication with my patients. | □ | □ | □ | □ | □ |
| [FC] m. | …be stimulated by my organization/manager. | □ | □ | □ | □ | □ |
| [EE] n. | …be fun to use. | □ | □ | □ | □ | □ |
| [PE] o. | …decrease the interaction/communication with my patients. | □ | □ | □ | □ | □ |
| [PE] p. | …increase the tailoring of care among my patients. | □ | □ | □ | □ | □ |
| [EE] q. | …ask a lot of new skills from me. | □ | □ | □ | □ | □ |
| [EE] r. | …are easy to gain skills in. | □ | □ | □ | □ | □ |
| [SI] s. | …be actively used by my colleagues. | □ | □ | □ | □ | □ |
| [EE] t. | …be interesting to use. | □ | □ | □ | □ | □ |
| [FC] u. | …fit within my way of working. | □ | □ | □ | □ | □ |
| [SI] v. | …be seen as a positive development by my organization/manager. | □ | □ | □ | □ | □ |
| [FC] w. | …fit with the goals of (the management of) my organization. | □ | □ | □ | □ | □ |
| [FC] x. | …fit with the possibilities I receive for schooling and education. | □ | □ | □ | □ | □ |
|  |  |  |  |  |  |  |

* PE = Performance expectancy

EE = Effort expectancy

SI = Social influence

FC = Facilitating conditions

|  |  | Totally disagree | Partly disagree | neutral | Partly agree | Totally agree |
| --- | --- | --- | --- | --- | --- | --- |
| 19. | I would like to use/keep using online self-management programs in my work. | □ | □ | □ | □ | □ |
| 20. | I intend to use/keep using online self  management programs in my work. | □ | □ | □ | □ | □ |
| 21. | I expect to use/keep using online self  management programs in my work. | □ | □ | □ | □ | □ |
| 22. | Within what time frame do you expect to use  online self-management programs in your  practice? | □ I currently use them already | | | | |
|  |  | □ Within the next six months | | | | |
|  |  | □ Within the next year | | | | |
|  |  | □ Within two to five years | | | | |
|  |  | □ Not within the next five years | | | | |
|  |  | □ Never | | | | |
